# Use of ammonium sulphate as a sulphur fertilizer: Implications for ammonia volatilization

**DOI:** 10.1111/sum.12733

**Published:** 2021-07-02

**Authors:** David S. Powlson, Chris J. Dawson

**Affiliations:** ^1^ Department of Sustainable Agriculture Systems Rothamsted Research Harpenden Herts UK; ^2^ Chris Dawson and Associates Westover York UK

**Keywords:** ammonia, ammonium sulphate, fertilizer, gothenburg convention, sulphur, volatilization

## Abstract

Ammonium sulphate is widely used as a sulphur (S) fertilizer, constituting about 50% of global S use. Within nitrogen (N) management, it is well known that ammonium‐based fertilizers are subject to ammonia (NH_3_) volatilization in soils with pH > 7, but this has been overlooked in decision making on S fertilization. We reviewed 41 publications reporting measurements of NH_3_ loss from ammonium sulphate in 16 countries covering a wide range of soil types and climates. In field experiments, loss was mostly <5% of applied N in soils with pH (in water) <7.0. In soils with pH > 7.0, there was a wide range of losses (0%–66%), with many in the 20%–40% range and some indication of increased loss (ca. 5%–15%) in soils with pH 6.5–7.0. We estimate that replacing ammonium sulphate with a different form of S for arable crops could decrease NH_3_ emissions from this source by 90%, even taking account of likely emissions from alternative fertilizers to replace the N, but chosen for low NH_3_ emission. For every kt of ammonium sulphate replaced on soils of pH > 7.0 in temperate regions, NH_3_ emission would decrease from 35.7 to 3.6 t NH_3_. Other readily available sources of S include single superphosphate, potassium sulphate, magnesium sulphate, calcium sulphate dihydrate (gypsum), and polyhalite (Polysulphate). In view of the large areas of high pH soils globally, this change of S fertilizer selection would make a significant contribution to decreasing NH_3_ emissions worldwide, contributing to necessary cuts to meet agreed ceilings under the Gothenburg Convention.

## 1 INTRODUCTION

It has been recognized for over 50 years that surface application of ammonium‐based fertilizers or urea can lead to rapid and significant evolution of ammonia (NH_3_) gas to the atmosphere (Gasser, 1964, and references therein). It is also well established that loss is greater in soils with pH > 7 and/or containing calcium carbonate. For example, in laboratory experiments Fenn and Kissel (1975) found that up to 50% of the nitrogen (N) applied as ammonium sulphate could be volatilized as NH_3_ depending on calcium carbonate content of the soil. Losses are often greater with urea because rapid conversion of urea‐N to ammonium‐N by the urease enzyme in soil increases pH in the vicinity of fertilizer particles (Rachhpal‐Singh & Nye, 1986; Kirk & Nye, 1991). These well‐established principles were summarized by Harrison and Webb (2001) in the context of comparing gaseous N losses from urea with those from ammonium nitrate and other forms of N fertilizer.

In addition to being a cause of decreased N use efficiency by crops, NH_3_ emission has adverse environmental and public health impacts, including the following:

1. Redeposition of NH_3_ on to soil or water causes nutrient enrichment, which is particularly damaging to the ecology of semi‐natural sites (Guthrie et al., 2018; Stevens et al., 2004).
2. Microbial nitrification of redeposited NH_3_ causes acidification of soil and water because the process produces protons and, thus, acidification of the environment (Goulding et al., 1998; Johnston et al., 1986).
3. Ammonia gas in the atmosphere can react with other substances to form particulate materials including ammonium sulphate, ammonium nitrate, and ammonium chloride. Human exposure to these particulates (PM10 and PM2.5) can lead to increased rates of respiratory and cardiovascular illness (Moldanová, et al., 2011; Wu et al., 2016).

All European countries (including the EU as a whole), plus several others including the USA, Canada, and Russia, are signatories to the UN Gothenburg Convention on Long‐range Transboundary Air Pollution: see UNECE (2015) for guidance on preventing and abating NH_3_ emissions from agricultural sources in accordance with the convention. These countries are therefore committed to decreasing emissions of NH_3_ and other pollutant gases. Agriculture is a major source of NH_3_, estimated at >90% of total emissions in the European Union in 2018 (EEA, 2020a) and 87% of UK emissions in 2018 (Defra, 2020). The majority of agricultural emissions are associated with manure, with 18% from fertilizers in the UK (Defra, 2020). Consequently, there is strong pressure to decrease agricultural emissions across much of the world.

In recent years, studies on NH_3_ emissions from agriculture have focused mainly on animal manure and urea because these are the major sources (Bouwman et al., 2002; Del Moro et al., 2017). Globally ammonium sulphate is a relatively minor contributor to N fertilizer use, global production being estimated as 5.67 Mt N in 2017 compared to 77.87 Mt N as urea and 16.11 Mt N as ammonium nitrate (IFA, 2017; internal data used with permission). However, in recent years ammonium sulphate has become a major source of sulphur (S) for fertilizer use because it is readily available, being a by‐product of various industrial processes, and has been relatively cheap compared to most other forms. Global use of S fertilizer in 2015 was reported as 13.3 Mt S (equivalent to 33.3 Mt SO_3_, the unit in which S fertilizer quantities are usually quoted in the context of production and agronomic use) of which about 50% was as ammonium sulphate, used either as the pure material, in blends with other straight N fertilizers or as part of compound NPKS fertilizers (IFA, 2017).

With ammonium sulphate being used more widely as a source of S for crops, it is inevitable that some NH_3_ will be volatilized, thus working against the aim of the Gothenburg Convention. Volatilization will be greatest from calcareous soils and others with a pH of 7 or greater. There are significant areas of such soils globally in places where there is high‐yielding agriculture, and where S fertilizer is either already widely used or its use is likely to increase. These include regions of China, India, Pakistan, the USA, France and UK. The aims of this paper are as follows: (1) to review data on NH_3_ emissions from ammonium sulphate; (2) estimate the decrease in NH_3_ emission achievable through a change to alternative sources of S. Such information is required as a basis for decisions regarding management practices including alternative sources of S, especially for top‐dressing on high pH and calcareous soils.

## 2 MATERIALS AND METHODS

We summarized the estimations of NH_3_ emission factors (EFs) for ammonium sulphate proposed in documents from several major regulatory authorities internationally and from previously published literature reviews. We then summarized results from experiments in which NH_3_ volatilization from ammonium sulphate has been measured in both laboratory and field experiments (Tables S1 and S2). This was based on a literature search using *Web of Science* during February to March 2019 using the search term “ammonium sulphate” or “ammonium sulphate” modified by “fertilizer” or “fertiliser” and “ammonia”. In most parts of the world ammonium sulphate is no longer widely used as an N fertilizer. However, in publications from the last 20 years or so, it is sometimes included for comparison with losses from urea or animal manures. We excluded publications where information on the soil type or environmental conditions was lacking or where the data on ammonium sulphate were non‐quantitative (e.g., NH_3_ volatilization simply stated as being less than that from urea). This review was informed by publications from 17 countries covering a wide range of climatic conditions, with 11 reporting results from laboratory experiments and 30 reporting from field experiments. Where publications report EFs for urea, we include these data for comparison. For the purposes of national reporting under the Gothenburg Convention, EFs are normally quoted as g NH_3_ evolved per kg N applied; this unit is used in Table 1 (taken from EEA, 2019) and in our estimations in Table 3 of the potential for decreasing NH_3_ emissions by changing S applications from ammonium sulphate to a different fertilizer form. However, in scientific studies of NH_3_ volatilization it is more usual to quote losses as the quantity of NH_3_‐N emitted as a percentage of N applied, so in our review of published data (Tables S1 and S2) we use these units.

**TABLE 1:** Emission factors (EFs) for ammonium sulphate, urea, and calcium ammonium nitrate (CAN) from EEA (2019). Ammonia emitted expressed as g NH_3_ per kg N applied (upper part of Table) and as NH_3_‐N emitted as percentage of N applied (lower part of Table)

N fertilizer form	Climate					
	Cool		Temperate		Warm	
	Soil pH					
	≤7.0	>7.0	≤7.0	>7.0	≤7.0	>7.0
	g NH_3_ per kg N applied					
Ammonium sulphate	90	165	92	170	115	212
Urea	155	164	159	168	198	210
CAN	8	17	8	17	10	21
	NH_3_‐N as % of N applied					
Ammonium sulphate	7.4	13.6	7.6	14.0	9.5	17.5
Urea	12.8	13.4	13.1	13.8	16.3	17.3
CAN	0.7	1.4	0.7	1.4	0.8	1.7

## 3 RESULTS

### 3.1 Emission factors from official and regulatory bodies

The United Nations Economic Commission for Europe (UNECE) Framework Code for Good Agricultural Practice for Reducing Ammonia Emissions (UNECE, 2015) does not state a specific EF for ammonium sulphate but includes the following statement: “On calcareous soils (pH > 7.5) do not use ammonium sulphate fertilizers if rapid incorporation, injection into the soil, immediate irrigation or the use of polymer‐coated fertilizer is not possible, but seek alternative sources of N and sulphur.” Similarly, the UK Code of Good Agricultural Practice for Reducing Ammonia Emissions (Defra, 2018), based in part on the model of Misselbrook et al. (2004), does not cite an EF for ammonium sulphate but states that, to minimize volatilization, surface application should be avoided on calcareous soil of pH > 7.5 unless it can be rapidly incorporated into soil.

The European Environment Agency (EEA) publishes technical guidance for preparing national emissions inventories for a range of atmospheric pollutants including NH_3_ (EEA, 2019). The guidance includes the EFs shown in Table 1 for Tier 2 level calculations for use in Europe and the wider UNECE geographical area. Values are expressed in units of g NH_3_ emitted per kg N applied (as published by EEA) in the upper part of the Table and converted to NH_3_‐N emitted as % of N applied in the lower part. The proposed values show three main trends. First, in agreement with other studies, soil pH has a large influence on NH_3_ volatilization from ammonium sulphate. For example, under temperate climatic conditions, the EF for soil with pH ≤ 7.0 is 7.6% of N applied compared with 14% at pH > 7.0 (changing from 92 to 170 g NH_3_ per kg N applied). Second, there is a modest influence of temperature with slightly increased EF values in warmer climates. Third, in soils of neutral pH or lower, volatilization from ammonium sulphate is markedly less than from urea, for example, in temperate climates, 7.6% of N applied compared to 13.1%. But in soils with pH >7.0, which are normally calcareous, the difference virtually disappears.

The US Environmental Protection Agency recommended EFs for ammonium sulphate and urea of 8% and 15% of N applied, respectively (i.e., 97 and 187 g NH_3_ per kg N applied, respectively; EPA, 1994). In this, they followed the values recommended by Asman (1992). It was noted that soil pH and clay content (taken as a proxy for cation exchange capacity) were factors influencing NH_3_ loss, but it was decided to give only a single EF value for each N fertilizer type.

### 3.2 Earlier reviews

Bouwman et al. (2002) reviewed published literature at that time on NH_3_ volatilization from fertilizers and manures as a basis for estimating the contribution of agriculture to global emissions. Although their data are not ideal for our current purpose, some general points emerge. First, based on about 150 publications, they concluded that laboratory measurements of NH_3_ volatilization gave values that were 47%–64% higher than field measurements. This is almost certainly because the commonly used laboratory techniques involve air being forced through an incubation vessel, removing NH_3_ from the soil atmosphere and stimulating further emission by altering equilibria in soil solution. Second, they concluded that the overall mean emissions factors were 18.7% of N applied for ammonium sulphate and 21% for urea, based on 86 data points. The corresponding median values were 11.2% and 14%, respectively. Third, their review showed an effect of soil pH, with EF increasing from 15% of N applied for soils with pH ≤ 5.5 to around 20% for soil with pH > 7.5. However, it should be emphasized that these latter values are means for all forms of N fertilizer, not specifically ammonium sulphate.

On the basis of a meta‐analysis of >800 publications concerning N fertilizer management, Pan et al. (2016) concluded that NH_3_ volatilization averaged 74% less from non‐urea based fertilizers compared to urea, though this is not in agreement with the findings of Bouwman et al. (2002). However, Pan et al. (2016) did not explicitly identify losses from ammonium sulphate.

### 3.3 Laboratory experiments

Results from 11 publications we reviewed giving results from laboratory experiments are summarized in Table S1. The earliest papers cited are Martin and Chapman (1951) and Gasser (1964). These authors refer to papers dating back to 1939, though they mainly refer to losses from urea. As with the earlier reviews, a clear conclusion is that soil pH and CaCO_3_ content both have a major influence on NH_3_ volatilization from applied ammonium sulphate, with high pH favoring greatly increased loss. This was shown in two ways: by comparing NH_3_ loss from soils that naturally differed in pH (e.g., Martin & Chapman, 1951; Whitehead & Raistrick, 1990) or by adjusting the pH of a single soil in the laboratory (He et al., 1999). In a well‐known paper, Whitehead and Raistrick (1990) applied ammonium sulphate, and other forms of N‐containing fertilizers, to the surface of a set of UK soils in columns and measured NH_3_ volatilization over 8 days. In a soil of pH 6.1 containing 0.6% CaCO_3_, emission from ammonium sulphate was small (4% of applied N; Table S1) but increased to 31% in a soil of pH 7.1 and containing 1.8% CaCO_3_. A similar trend with increasing soil pH was seen in soils from the USA (Liu et al., 2007; Martin & Chapman, 1951) and Kenya (Siguna et al., 2002). He et al. (1999) took a soil from Florida of pH 7.9 and adjusted pH by adding HCl or NaOH. When soil pH was below 5.5, there was no measurable volatilization of NH_3_ from ammonium sulphate, but when adjusted to pH 6.5 or above, emission was around 30% of applied N (Table S1). On the basis of his own and earlier studies, Gasser (1964) noted that there was a close relationship between NH_3_ loss and soil cation exchange capacity (CEC), but later authors state that pH has a much stronger effect.

**FIGURE 1:**
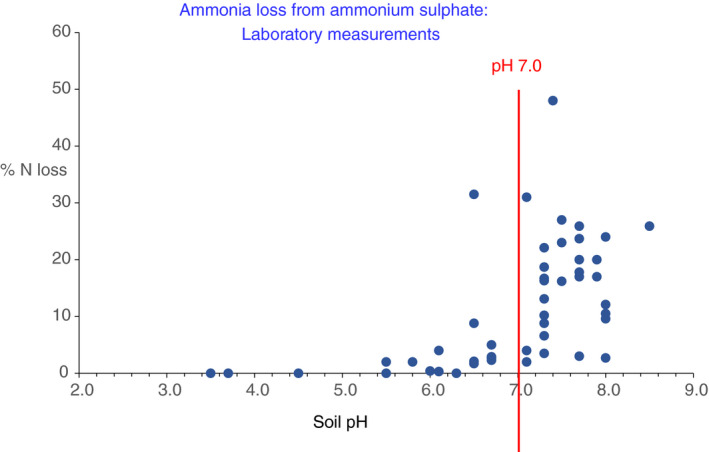
Influence of soil pH on NH_3_ emission from ammonium sulphate. Emission from urea included, for comparison, if included in reviewed literature article. Data from laboratory experiments

A sharp increase in the likelihood of substantial NH_3_ loss as soil pH exceeds 7.0 is clearly shown in Figure 1: With only one exception, losses from soil with pH < 7.0 were <10%, and mostly <5% of the N applied as ammonium sulphate. In soils of pH > 7.0, losses were very variable but with many at 20% or higher. In the one example of a large loss from a soil with pH < 7.0 (a 32% loss from a soil at pH 6.5; He et al., 1999), the authors noted that nitrification was unusually slow in this soil, which had been adjusted to this pH from its natural value of 7.9; N remained in the ammonium form for longer than in the soils adjusted to pH 7.5 or 8.5, which gave a slightly smaller loss (Table S1). This longer persistence of ammonium‐N in a soil with artificially adjusted pH almost certainly permitted a greater conversion of N to NH_3_ and its subsequent gaseous loss and is unlikely to be relevant to practical field situations.

Ammonia volatilization from ammonium sulphate generally increases at higher temperature as shown by a comparison of EF at 22 and 32℃ in soil from Trinidad (Prasad, 1976). Soil moisture is also an influencing factor, with wetter conditions tending to decrease loss (Table S1; Liu et al., 2007; Prasad, 1976). The physical and chemical processes in soil, especially pH effects and the presence of CaCO_3_, that influence equilibration between NH_4_^+^ ions and NH_3_ and determine the rate of NH_3_ diffusion through soil and loss to the atmosphere have been well understood for many years (Fenn & Kissel, 1973, 1975; Fenn & Hossner, 1985; Rachhpal‐Singh & Nye, 1986; Harrison & Webb, 2001). As expected, where surface application was compared with ammonium sulphate mixed with the soil (Gasser, 1964), mixing decreased volatilization somewhat.

In several cases, though not all, volatilization from ammonium sulphate was less than that from urea under the same conditions (Liu et al., 2007; Prasad, 1976; Shahandeh et al., 1992; Whitehead & Raistrick, 1990; Table S1). This is because of the well‐known effect of urea hydrolysis causing an increase in soil solution pH in the vicinity of fertilizer granules (Rachhpal‐Singh & Nye, 1986; Rochette et al., 2009). A result of this is that volatilization from urea can occur in soils that have a more acidic pH. One example is in the data of Whitehead and Raistrick (1990), where soil pH was 6.1 or 5.5 NH_3_ volatilization from ammonium sulphate was negligible but from urea was 38% and 22% of applied N, respectively.

### 3.4 Field experiments

Data from 30 publications showing results from field experiments are summarized in Table S2. In most studies, the main focus was NH_3_ volatilization from urea, with ammonium sulphate being included as a comparison and expected to give a smaller loss. Where there are data from a urea treatment under equivalent conditions, these are included. The studies are from 11 countries with climates ranging from cool temperate (including the USA, UK, Denmark) to tropical with climates that are low rainfall (eg, Syria, Sudan) or higher rainfall (Brazil).

A wide range of measurement methods was used. In the majority of cases, it was some form of semi‐open chamber such that air in a chamber inserted into soil could exchange with the atmosphere via a filter impregnated with acid in order to trap NH_3_, which was then quantitatively determined. In a few cases, there was an arrangement for scrubbed air to flow through the chambers prior to absorption of NH_3_, and in some earlier studies, completely closed chambers were used (Musa, 1968; Volk, 1959). In some, micrometeorology was used (Hayashi et al., 2011; Huo et al., 2015; Turner et al., 2012), and in five cases, NH_3_ volatilization was calculated from ^15^N recovery in situations where it was deduced that other N loss processes were small (Fenilli et al., 2008; Isa et al., 2006; Malhi et al., 1996; Pilbeam et al., 1997; Pilbeam & Hutchison, 1998). Wind tunnels, which are widely used for measuring NH_3_ loss from manures and urea, were only used in one of the studies reported in Table S2 (Sommer & Jensen, 1994). In addition to the influence of soil type, cropping system and climate, and variability due to the range of measurement methods, field results are obviously affected by method of application, agronomic factors, and local weather conditions at the time of the experiment.

Interestingly, 10 publications were from Brazil where it appears that ammonium sulphate is more widely used as an N source than in many other regions. At all Brazilian sites, soil pH was acidic, ranging from 4.4 to 5.8, and in almost all cases, NH_3_ volatilization was small: <12% of N applied and mostly 0%–5%. By contrast, volatilization from urea was often considerably greater, ranging from negligible to >40%, presumably because soil pH was increased locally by urea hydrolysis.

**FIGURE 2:**
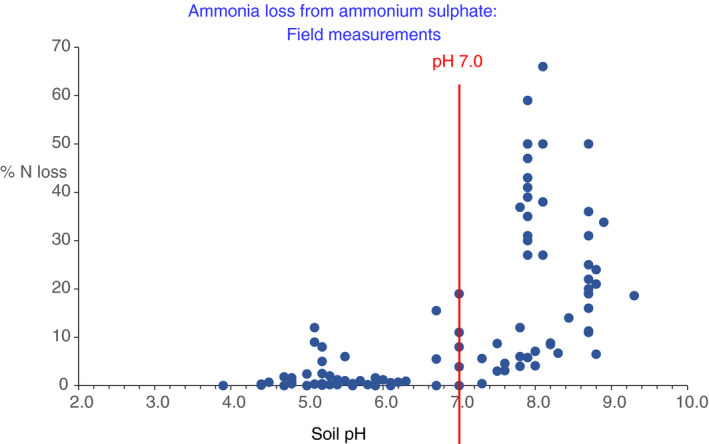
Influence of soil pH on NH_3_ emission from ammonium sulphate. Emission from urea included, for comparison, if included in reviewed literature article. Data from field experiments

As with the data from laboratory studies, Figure 2 shows that soil pH has a dominant influence on NH_3_ volatilization from ammonium sulphate under field conditions. Losses of >20% of applied N were all associated with soil pH > 7.3 (Figure 2). The largest losses of 27–66% were at sites in Syria with soil pH 8.1 and 23% CaCO_3_ (Pilbeam & Hutchinson, 1998; Pilbeam et al., 1997), Sudan (soil pH 8.7 with 4% CaCO_3_; Musa, 1968), and the USA (soil pH 7.6 – 8.2, with 25% CaCO_3_; Hargrove et al., 1977). However, intermediate losses (up to approx. 20% of N applied) were recorded at sites with soil pH values between 6.7 and 7.3 (Figure 2). In one set of experiments in Australia, with soil pH around 7.7, whether soils were described as having “low” or “high” calcium carbonate content made the difference between losses of <10% or 20%–35% (Schwenke et al., 2014; Table S2). At a site in Tanzania (Isa et al., 2006), soil salinity was associated with higher pH and increased NH_3_ volatilization. Results from laboratory experiments showed the same trend of increased NH_3_ loss at soil pH of 7.0 or a little below. Hargrove et al. (1977) noted that the measured losses (33%–41% of N applied) from soils of pH 7.6 to 8.2 under pasture in the USA were influenced by temperature at the time of application. Martha et al. (2004) found a similar trend in Brazil.

## 4 DISCUSSION

### 4.1 Effect of soil factors on ammonia loss from ammonium sulphate

Results from laboratory and field studies clearly show that soil pH, together with calcium carbonate content, is the overriding factor determining NH_3_ emission from applied ammonium sulphate. In field experiments, where soil pH was below 7, N lost as NH_3_ was well below 5% of N applied in the majority of cases (Figure 2). Where soil pH was between 6.5 and 7.0, losses of 5%–15% were observed and 5%–10% in a few cases at lower pH. For soils with pH 7.0 or greater, losses of 15%–35% of N applied were commonly observed with 50% or more in some cases (Figure 2). However, there were also a few cases where losses were below 10%, even where pH was between 7.0 and 8.0; the reason is not known but is likely to be associated with the precise details of application method in relation to crop growth and weather conditions. For example, rainfall soon after application, rapid nitrification of ammonium, or rapid crop uptake of N would all decrease the possibility of NH_3_ volatilization.

A clear conclusion for S fertilization practice is that it is inadvisable to apply ammonium sulphate to soils with pH above 7.0 because NH_3_ volatilization is extremely likely to be significant; even in soils with pH between 6 and 7, there is some risk of loss. By contrast, in soil of lower pH the risk is small. These general trends are in line with the EFs proposed by the EEA (Table 1). However, about half of the data points in Figure 2 for soils with pH > 7.0 indicate EFs greater than the 14%–18% range proposed by EEA, in several cases considerably greater: We can offer no explanation for this. The use of average EFs defined for wide soil pH ranges and climate categories represents a broad and pragmatic generalization. The actual loss of NH_3_ in any specific situation will be determined by specific agronomic conditions and environmental factors, including weather around the time of ammonium sulphate application. In addition, the reported losses shown in Figure 2 will also be influenced by the different methods of measurement used. For these reasons, it was concluded that detailed statistical analysis of the data was unlikely to be helpful in further identifying the relative importance of different factors influencing NH_3_ loss.

### 4.2 Global implications for sulphur fertilization using ammonium sulphate

These conclusions are extremely pertinent when considering the use of ammonium sulphate as a source of S for arable crops. Many arable soils, especially in temperate climatic zones, are limed in order to maintain a pH of about 7. In addition, significant areas of soil are naturally calcareous. This is illustrated for the UK by analyses of soils from farmers’ fields conducted by professional laboratories as part of routine soil testing for fertilizer advice (PAAG, 2019). Table 2 shows mean data over 10 years, based on >1.5 million samples.

Within arable soils, 40% had pH > 7.0, 21% between 6.5 and 7.0 and a further 21% between 6.0 and 6.5 (Table 2). Thus, based on this large sample of arable fields, 40% were at a pH likely to lead to NH_3_ losses of 15–35% of applied N, with a risk of 50% loss in some cases. In addition, a further 42% of fields were in the pH range 6.0–7.0, with a possibility of around 10% loss. The risk of substantial loss from grassland fields is less as only 8% of samples analyzed were at pH > 7.0 (Table 2).

**TABLE 2:** Soil pH of UK agricultural soils. Based on >1,500,000 samples analyzed over the 10‐year period 2009/10 to 2018/19, as reported by PAAG (2019)

	Percentage of samples in class–10‐year average							
	pH							
	<5.00	5.00–5.49	5.50–5.99	6.00–6.49	6.50–6.99	7.00–7.49	7.50–7.99	>8.00
Arable	1	5	13	21	21	16	16	8
Grass	2	18	36	26	11	4	3	1

**TABLE 3:** Estimation of NH_3_ emission from application of ammonium sulphate as an S source to soils of pH > 7.0 and <7.0 and potential decrease from replacing the N supplied from ammonium sulphate by calcium ammonium nitrate (CAN). Calculated for a unit 1 kt ammonium sulphate

Item	Unit	Climate			
		Temperate		Warm	
Soil pH_aq_		>7.0	<7.0	>7.0	>7.0
Per kt ammonium sulphate					
Nitrogen (N) content of 1 kt ammonium sulphate	t N	210	210	210	210
NH_3_ emission factor for ammonium sulphate on soils of different pH (EEA 2019)	g NH_3_ per kg N	170	92	212	115
Total potential emission of NH_3_ from use of ammonium sulphate to supply required nutrient sulphur	t NH_3_	35.7	19.3	44.5	24.2
NH_3_ emission factor for CAN on soils of different pH (EEA 2019)	g NH_3_ per kg N	17	8	21	10
Total potential emission of NH_3_ from CAN used as replacement for the N from ammonium sulphate	t NH_3_	3.6	1.7	4.4	2.1
NH_3_ emission reduction from replacing ammonium sulphate with CAN plus a zero‐N sulphur source	t NH_3_	32.1	17.6	40.1	22.1
Potential percent reduction in NH3 emissions from replacement of ammonium sulphate by CAN	%	90.0	91.3	90.1	91.3

**FIGURE 3:**
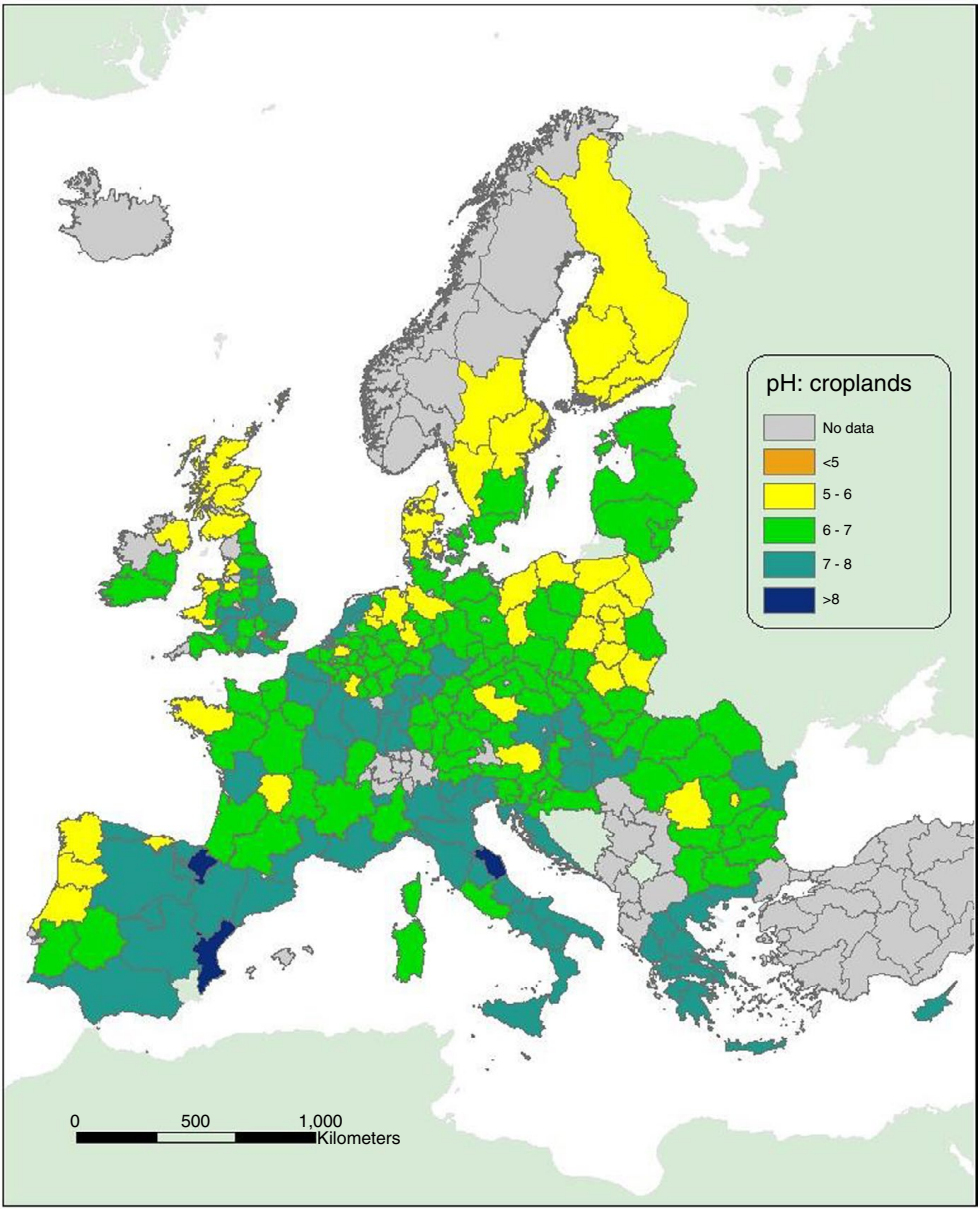
Topsoil pH (in water) in croplands within Europe. Derived from LUCAS 2015 topsoil survey, JRC Technical Report, EU, 2020 (Jones et al., 2020)

For Europe as a whole, many major arable cropping areas have soil of high pH. Figure 3 (taken from Jones et al., 2020) shows soil pH (in water) for croplands, based on the LUCAS database and illustrated for regions within the European Union at the level of NUTS 2 (Nomenclature of Territorial Units for Statistics; see Jones et al. (2020) for full description). Of the 238 regions where there were sufficient data for cropland soil properties to be illustrated in this way, over 30% had soil pH > 7. In addition to much of southern and eastern England, substantial areas of northern and central France are in this category. Southeast England and northern France, including the Paris Basin, are both important regions for cereal and oilseed production where S fertilizers are widely used and dressing cover is likely to increase (Webb et al., 2016). In northern Europe, Figure 3 also shows that significant areas used for arable cropping in Germany, Hungary, and the Netherlands have soils in this pH category and thus with a high risk of NH_3_ emission if S is supplied as ammonium sulphate. Further south, large areas in Spain, southern France, Italy, Croatia, Greece, and Romania are also in this category. Across Europe, a similar additional area of cropland is in the pH 6–7 category; within this, there are significant areas with soil pH > 6.5 and thus at some risk of NH_3_ emission; see Ballabio et al. (2019) for a soil pH map derived from the LUCAS data using Gaussian process regression modelling.

Globally, many major agricultural regions include substantial areas of soil with pH > 7.0 and/or large calcium carbonate concentrations. The Indo‐Gangetic Plain in India, Pakistan, Nepal, and Bangladesh is an extremely important agricultural region, with much intensive arable cropping, and significant areas with high soil pH and calcium carbonate content and, in some cases, sodic conditions (e.g., Pal et al., 2009). In China, although there is a widespread problem of soil acidification, a recent mapping study (Chen et al., 2019) also showed many soils with pH > 7.0 including a significant number at around pH 8.0. These were mainly located in northern and western China including the North China Plain that is important for wheat and maize production, but also includes the karst region in southwest China, covering 540,000 km^2^ (Wang et al., 2019). In both India and China, there is widespread S deficiency in crops and increasing quantities of S fertilizers are being used. In both countries, especially in the Indo‐Gangetic Plain and the North China Plain, high rates of N fertilizer are used with the aim of achieving large crop yields, so the requirement for S will almost certainly increase further. Many soils in Turkey have high pH and high Ca content; for example, Gezgin and Bayrakll (1995) measured NH_3_ losses from ammonium sulphate of 14%–20% from a soil with pH 8.44 and which contained 20% CaCO_3_ (Table S2).

### 4.3 Estimating potential for decreasing ammonia emission by replacing ammonium sulphate with alternative fertilizers as a source of sulphur

Table 3 shows an estimation of the potential for decreased NH_3_ emissions if ammonium sulphate was replaced by an alternative source of S, not prone to NH_3_ volatilization. The calculations are made for a unit of 1 kt of ammonium sulphate, so the resulting values can be applied generically to any region. For the purposes of this estimation, we assume that all ammonium sulphate is applied to the soil surface (i.e., top‐dressed). In this estimation, we use the EF values from EEA (2019; Table 1) even though, as discussed above, there was a suggestion from our review of data (Figure 2) that EFs could often be greater. We therefore suggest that the values we derive for decreased NH_3_ emission are conservative. The estimation is made for soils having a pH of pH > 7.0 and those with pH < 7.0 in both temperate and warm climates. We make calculations using EFs expressed as g NH_3_ kg^−1^ N applied because this is the unit most commonly used in national inventories. Table 3 indicates that applying 1 kt of ammonium sulphate to soil with pH > 7.0 leads to emissions of 35.7 and 44.5 t NH_3_ in temperate and warm climatic regions, respectively; in principle, these emissions could be completely eliminated if ammonium sulphate was to be replaced as the source of S. However, the N supplied by ammonium sulphate would need to be replaced, almost certainly leading to some emission of NH_3_ and offsetting this reduction. Obviously, there would be no benefit from using urea as the source of N as its NH_3_ EF is generally greater than that of ammonium sulphate. For the purposes of this calculation, we assume the N is replaced by calcium ammonium nitrate (CAN), an N fertilizer with a low EF for NH_3_. Emissions from CAN, to replace the N previously supplied from ammonium sulphate, are estimated as 3.6 and 4.4 t NH_3_ per kt N for soils of pH > 7.0 in temperate and warm climates, respectively, about 10% of the emissions from ammonium sulphate. Hence, the overall benefits from this change are still substantial for soils of pH > 7.0: decreases of 32.1 t NH_3_ (temperate climate) and 40.1 t NH_3_ (warm climate) per kt ammonium sulphate replaced. The corresponding reductions for lower pH soils are 17.7 and 22.1 t NH_3_ per kt ammonium sulphate replaced. On all soils, these represent decreases in NH_3_ emission of over 90% compared to using ammonium sulphate (Table 3).

For any country or region, the absolute reduction in NH_3_ emissions possible through a change away from using ammonium sulphate as the source of S will depend on (a) the total usage of ammonium sulphate for the region and (b) the proportion that is applied to soils of pH > 7.0; in most cases, specific data on the latter value are not available, so indirect deductions are necessary. Table 4 shows the annual usage of ammonium sulphate in a range of countries; the largest usages globally (>1,000 kt per year) being in Brazil, the USA, Indonesia, Mexico and Vietnam. Within Europe, Germany, Spain and UK are the largest users; in these countries, it is likely that the majority is used as a source of S. All three countries, and many others in the EU, need to decrease NH_3_ emissions immediately by up to 10% to meet the lowered ceilings introduced under the Gothenburg Conventions for 2020 and by up to 20% to meet the planned ceilings for 2030 (Table 5). Although the largest decreases are likely to be achieved by improved management of manure, or of urea fertilizer where this is the dominant form of N fertilizer, any additional savings will be beneficial and the alteration in S fertilizer use discussed here is relatively easy to achieve.

**TABLE 4:** Consumption of ammonium sulphate by country in 2017, kt product. Data from IFA (2020)

Country	kt product
Brazil	1,999
U.S.A.	1,919
Indonesia	1,840
Mexico	1,096
Viet Nam	1,052
Canada	823
Malaysia	745
Turkey	727
Philippines	583
India	563
Thailand	526
Germany	510
Ukraine	462
Australia	424
Spain	348
China	311
Japan	278
Russian Federation	271
Peru	250
United Kingdom	238
Egypt	231
Lithuania	215

**TABLE 5:** Percentage ammonia emission reductions required by EU Member States and the UK to meet 2020 and 2030 emission reduction commitments. Based on emissions in 2018. Data from EEA (2020b)

	2020	2030
Austria	x	xx
Belgium	✓	x
Bulgaria	✓	x
Croatia	✓	x
Cyprus	x	xx
Czechia	✓	xx
Denmark	xx	xx
Estonia	✓	✓
Finland	x	x
France	x	xx
Germany	x	xx
Greece	✓	✓
Hungary	x	xxx
Ireland	x	x
Italy	✓	x
Latvia	x	x
Lithuania	xx	xx
Luxembourg	✓	xx
Malta	✓	✓
Netherlands	✓	x
Poland	✓	xx
Portugal	✓	x
Romania	✓	xx
Slovakia	✓	xx
Slovenia	✓	x
Spain	x	xx
Sweden	x	x
United Kingdom	x	xx

✓, Current emission levels below the emission reduction commitment; x, Emission reduction needed by <10% from current levels; xx, Emission reduction needed by 10%–30% from current levels; xxx, Emission reduction needed by 30%–50% from current levels.

As an example of the benefit from making this change, total annual consumption of fertilizer S in the UK in 2017/18 was 90 kt S (AIC, 2019). It is estimated that at least 90% of this was provided as ammonium sulphate, and that 70% of this quantity was applied with other N fertilizers as a topdressing on arable land. Table 2 indicates that 40% of the UK arable land has a pH > 7.0 so that using the estimations in Table 3 it is calculated that the annual NH_3_ emission from this source in high pH arable land would be reduced by over 3 kt NH_3_ by changing to a different source of S, that is, almost 20% of the 16 kt decrease required to meet the 2020 ceiling.

In the global context, the relevant land area of the UK is small. In regions with large areas of high pH soils, as discussed earlier, the absolute decreases in NH_3_ emission possible will be considerably greater and would thus make a significant contribution to decreasing global emissions. With goals of increased crop yields and quality in many regions, achieved in part through application of N and other fertilizers, the requirement for S fertilizers will continue to increase, as will the focus on different sources of S and their various advantages and disadvantages.

### 4.4 Implications for appropriate choice of S fertilizers

For many soils types and environments, ammonium sulphate is a largely satisfactory source of S and has the advantage of simultaneously supplying part of the crop N requirement, but this analysis has strongly emphasized that it is highly undesirable to use it on soils with pH > 7.0, or even slightly lower. Although the risk of NH_3_ volatilization from ammonium‐based fertilizers has long been recognized in the context of N fertilizer use, it appears to have been overlooked in the context of selecting an appropriate S‐supplying fertilizer suitable for different soil types. It is common practice to surface‐apply S during the period of rapid crop growth, often together with at least part of the N application. Consequently, practices that could decrease NH_3_ volatilization from ammonium sulphate, such as incorporation into soil, are not feasible.

Several alternative sources of S, without any associated N, are available that would be preferable on high pH soils. These include potassium sulphate (often referred to as SOP, abbreviation for sulphate of potash), magnesium sulphate (kieserite), polyhalite (also known as polysulphate, a mineral containing sulphates of potassium, calcium, and magnesium), calcium sulphate dihydrate (gypsum), and single superphosphate SSP (comprising a mixture of monocalcium phosphate and gypsum). Obviously, with each of these S fertilizer materials, the content of P, K, Mg, or Ca needs to be taken into account when deciding on other nutrient applications. Elemental S can also be used but is more slowly available to crops than the other forms because it first has to be oxidized to sulphate by soil bacteria and the rate of conversion is somewhat unpredictable (e.g., Malhi et al., 2005; McGrath et al., 2002). Because several alternatives to ammonium sulphate are readily available and cost‐effective, replacing it by one of these, at least on soils of pH 7.0 or higher (and perhaps also on soils in the pH range 6.5–7.0) is a relatively easy change in agronomic practice that would make a significant contribution to reducing NH_3_ emissions in many countries as required for compliance with the Gothenburg Convention.

## Supporting information

Table S1

Table S2

## Data Availability

The data that supports the findings of this study are available in the supplementary material of this article.
