# Supplementary material for: Use of ammonium sulphate as a sulphur fertilizer: Implications for ammonia volatilization
Source: Soil Use Manag. 2021 Jul 2;38(1):622–34. doi: 10.1111/sum.12733 (PMC9290479; doi:10.1111/sum.12733)
Supplement: Supplementary file 1 — Table S1 [file SUM-38-622-s001.doc]

# Supplementary Table 1. Ammonia losses from ammonium sulphate: measurements under laboratory conditions.

| Reference | **Country** | **Soil pH** | **% CaCO_3_ In soil** | **Temperature, °C** | **Soil moisture status** | **Period (days)** | **Application method** | **N lost as NH_3_, % of N applied** | | **Measurement method** |
| --- | --- | --- | --- | --- | --- | --- | --- | --- | --- | --- |
|  |  |  |  |  |  |  |  | **Ammonium sulphate** | **Urea** |  |
| Gasser  (1964) | UK | 8.0 | 78 | 5 | 40% WHC | 41 | Surface | 12.1 | 10.6 | Sealed jars, flowing air |
|  |  |  |  |  |  |  | Mixed | 10.5 | 4.1 |  |
|  |  | 8.0 | 78 | 5 | 40% WHC | 41 | Surface | 9.6 | 6.8 |  |
|  |  |  |  |  |  |  | Mixed | 2.7 | 3.2 |  |
| Hamid &  Mahler (1994) | USA | 5.8 |  |  |  | 16 |  | 2 | 18 | Surface applied.  Closed containers, flowing air |
|  |  | 7.1 |  |  |  |  |  | 4 | 38 |  |
|  |  | 7.7 |  |  |  |  |  | 3 | 5 |  |
| He *et al.*  (1999) | USA | 7.9 |  | 30 | 90% WHC | 65 | Surface | 20 |  | Open vessels with acid treated sponge trap |
|  |  | 3.5 |  | 30 | 90% WHC | 21 | Surface | 0 |  | Open vessels with sponge trap treated with acid. pH adjusted |
|  |  | 4.5 |  |  |  |  |  | 0 |  |  |
|  |  | 5.5 |  |  |  |  |  | 0 |  |  |
|  |  | 6.5 |  |  |  |  |  | 31.5 |  |  |
|  |  | 7.5 |  |  |  |  |  | 27.0 |  |  |
|  |  | 8.5 |  |  |  |  |  | 25.9 |  |  |
| Liu *et al.*  (2007) | USA | 6.49-7.0 |  |  | 20% FC | 28 | Surface | 16-25 | 9-24 | Sponge trap with acid.  Temp. 11, 20, or 29°C – according to mean annual temp at sites |
|  |  |  |  |  | 80% FC |  |  | 4-9 | 3-23 |  |
|  |  | 7.1-7.27 |  |  | 20% FC | 28 | Surface | 0-8 | 11-16 |  |
|  |  |  |  |  | 80% FC |  |  | 0-4 | 3-16 |  |
| Martin & Chapman (1951) | USA | 6.7 | 0-2.5 |  |  | 70 | Surface | 5 | 36 | Air flowing through bottle to acid trap |
|  |  | 7.1 |  |  |  |  |  | 2 | 18 |  |
|  |  | 7.5 |  |  |  |  |  | 23 | 16 |  |
|  |  | 7.7 |  |  |  |  |  | 17 | 14 |  |
|  |  | 8.0 |  |  |  |  |  | 24 | 16 |  |
| Matsushima *et al*. (2009) | S. Korea | 6.3 |  |  |  | 30 |  | 0 | 1 | Plots in glasshouse. Closed traps. 3 periods of 10 days measurements following each N application |
| Prasad (1976) | Trinidad | 7.3 | 0.2 | 22 | Low | 21 |  | 10.2 | 11.1 | Flowing air |
|  |  |  |  |  | Med |  |  | 8.8 | 7.7 |  |
|  |  |  |  |  | High |  |  | 3.5 | 4.2 |  |
|  |  | 7.3 | 0.2 | 32 | Low | 21 |  | 16.7 | 20.6 |  |
|  |  |  |  |  | Med |  |  | 13.1 | 14.5 |  |
|  |  |  |  |  | High |  |  | 6.6 | 8.4 |  |
| Shahandeh et al (1992) | USA | 6.8  6.8  5.5  5.5 |  | 25 | 15  15  14  14 | 16 | Bare soil  Straw covered  Bare soil  Straw covered | 0.8  1.0  0.2  0.5 | 13.5  14.9  18.9  16.9 | Static chambers capped with acid-treated sponge |
| Siguna *et al.* (2002) | Kenya | 6.0 |  | 20 |  | 8 | Surface | 0.4 |  | Closed chambers |
|  |  | 6.1 |  |  |  |  |  | 0.3 |  |  |
|  |  | 7.5 |  |  |  |  |  | 16.2 |  |  |
|  |  | 7.7 |  |  |  |  |  | 17.8 |  |  |
| Whitehead & Raistrick (1990) | UK | 3.7 | 0 | 20 | 60% WHC | 8 | Surface | 0 | 0 | Soil in columns. Columns in jars with flowing air |
|  |  | 5.5 | 0 |  |  |  |  | 2 | 22 |  |
|  |  | 6.1 | 0.6 |  |  |  |  | 4 | 38 |  |
|  |  | 7.1 | 1.8 |  |  |  |  | 31 | 26 |  |
|  |  | 7.4 | 2.76 |  |  |  |  | 48 | 42 |  |
| Zia *et al*.  (1999) | Pakistan | 7.9 |  | 30 |  | 8 | Mixed | 17 |  | Closed chambers with boric acid traps, changed 6 times during incubation |
