# Supplementary material for: Use of ammonium sulphate as a sulphur fertilizer: Implications for ammonia volatilization
Source: Soil Use Manag. 2021 Jul 2;38(1):622–34. doi: 10.1111/sum.12733 (PMC9290479; doi:10.1111/sum.12733)
Supplement: Supplementary file 2 — Table S2 [file SUM-38-622-s002.doc]

# Supplementary Table 2. Ammonia losses from ammonium sulphate: measurements under field conditions

| **Reference** | **Country** | **Surface soil pH** | **% CaCO_3_ in soil** | **Cropping** | **Measurement period** | **N lost as NH_3_ from ammonium sulphate,  % of N applied** | **N lost as NH_3_ from urea,,  % of N applied** | **Method of measurement** |
| --- | --- | --- | --- | --- | --- | --- | --- | --- |
| Cantarella *et al..* (2008) | Brazil | 4.4 – 5.8 | - | Sugarcane.  Residues on surface | 40 days | 0 | 1-25% depending on weather | Semi-open chambers |
| Debreczeni & Berecz (1998) | Hungary | 6.6 – 6.8 | - | Winter wheat | 108 days | 0 |  | Closed chambers |
| Del Moro *et al.* (2017) | USA | 6.7 | 2 | Fallow | 34 days | 14 - 17 | 46 | Micromet |
| De Souza *et al.* (2017) | Brazil | 5.7 | - | Maize | 53 | 1 | >35 | Semi-open chambers |
| do Nascimento *et al.* (2013) | Brazil | 4.7 | - | Sugarcane.  Residues on surface | 22 | 0 | 9 | Semi-open chambers |
| Fenilli *et al.* (2008) | Brazil | 5.3 | - | Coffee | 1 yr | 2 |  | Semi-open chambers  Combined with ^15^N labelling |
| Fontoura & Bayer (2010) | Brazil | 4.6 – 5.2 | - | Maize | 20 days | 2  (mean of 4 seasons) | 1-25%  depending on weather | Semi-open chambers |
| Freney *et al.* (1992) | Australia | 4.8 – 7.3  (trash pH: 5.6 – 6.5) | - | Sugarcane.  Residues on surface | 40 days | 0.4 – 1.8 | 17-38 | Micromet |
| Gezgin & Bayrakll (1995) | Turkey | 8.44 | 20 | Winter wheat | 10 days | 14 – 20  Depending on N rate applied | 4 - 12 | Closed chamber |
| Hargrove *et al.* (1977) | USA | 7.6 – 8.2 | 25 | Pasture | 4-8 days | 33 – 41 Influenced by temperature at time of application |  | Chambers with flowing air. |
| Hayashi *et al.* (2011) | Japan | 5.6, Andosol | - | Wheat | 10 days | <1% |  | Micromet and dynamic chambers |
| Hayashi *et al.* (2009) | Japan | 5.9 | - | Komatsuna (Japanese mustard spinach). | 25 days total over 3 periods | 0 |  | Dynamic chambers with field lysimeters |
| Huo *et al.* (2015) | China | 8.2 |  | Winter wheat | 74 days | 8.5 | 12 | Micromet |
| Isa *et al.* (2006) | Tanzania | 7.8, (non-saline)  8.8, (saline) |  | Sugarcane. | 1 year, repeated.  Results mean of 2 years | 5  23 | 8  65 | ^15^N recovery |
| Lara Cabezas *et al* (2008) | Brazil | 5.2 (in CaCl_2_) |  | Maize | 51 days | 8 | 77 | Chambers |
| Li *et al.* (2019) | China | 5.5 |  | Maize | 3 periods of approx. 10 days following 3 split applications | 6 |  | Vented chambers |
| Malhi *et al.* (1996) | Canada | 7.0 |  | Spring barley | Growing season | *Site 1:* Zero till: 19  Conv till: 8  *Site 2:* Zero till: 11  Conv till: 0 | 21 5  23 0 | ^15^N recovery.  Deduced that other N losses were minimaL |

# Table 3. Ammonia losses from ammonium sulphate: measurements under field conditions (cont.)

| Reference | **Country** | **Surface soil pH** | **% CaCO_3_ in soil** | **Cropping** | **Measurement period** | **N lost as NH_3_ from ammonium sulphate,  % of N applied** | **N lost as NH_3_ from urea,,  % of N applied** | **Method of measurement** |
| --- | --- | --- | --- | --- | --- | --- | --- | --- |
| Marchesan *et al.* (2011) | Brazil | 5.0 |  | Irrigated rice |  | Negligible | Urea slightly greater |  |
| Martha *et al.* (2004) | Brazil | 5.8 |  | Elephant grass pasture | 18 days | 9 – 12  Influenced by time of application | 33-45 (higher temp at time of application)  9 (lower temp) | Semi-open chambers |
| Martins *et al.* (2015) | Brazil | 5.2 |  | No-till maize | 37 | 5 | 6 | Semi-open chambers. Calibrated by comparison with ^15^N recovery. |
| Musa (1968) | Sudan | 8.7 | 4 | Bare soil | 6 weeks | 10 – 49 Largest loss with frequent drying and rewetting |  | Closed chambers |
| Norman *et al.* (2009) | USA | 7.3 |  | Dry-seeded rice,  then flooded. | 20 days | 5-6 | 21 – 26  Influenced by period of delay between seeding and flooding | Semi-open chamber |
| Pilbeam *et al.* (1997) | Syria | 8.1 | 23 | Wheat | Growing season | Year 1 51  Year 2 66 | 66 | ^15^N recovery. Deduced that other N losses were small |
| Pilbeam & Hutchison (1998) | Syria | 8.1 | 23 | Bare soil cores under field conditions | 13 days | 27 | 49 | ^15^N recovery. Deduced that other N losses were small |
| Schwenke *et al.* (2014) | Australia | 7.0 – 8.8 | 0.1 – 12.7 | Fallow | 1 month | Low CaCO_3_ 6 (3-11)  High (>10%) CaCO_3_ 26 (19-34) | 11 (5-19) | Micromet |
|  |  |  |  | Pasture |  | Low CaCO_3_ 6 (4-9) | 22-3 |  |
| Sommer & Jensen (1994) | Denmark | 6.1 |  | Wheat and grassland | 20 days | <2 | 25 | Wind tunnels |
| Thyssen *et al.* (2006) | Canada | 3.9 -5.1 |  | Wild blueberry | 12 days | Mean of 4 sites: <1 | 4 | Vented chambers |
| Turner *et al.* (2012) | Australia | 7.8  7.5 |  | Wheat  Barley | 10-18 days | 12  3 | 22  12 | Micromet |
| Viero *et al.* (2014) | Brazil | 5.4 – 6.1 |  | Winter wheat  Summer maize | 21 days | 2 | 2-15%  (Higher in summer) | Semi-open chambers |
| Volk (1959) | USA | 4.4 – 7.8 |  | Bare soil | 7 days | pH 6.7 5.5  pH 7.8 36.9  pH 4.4 – 6.3 <2 | 15.2  14.4  2-59 | Closed chambers |
